# Mechanisms of Impaired Lung Development and Ciliation in Mannosidase-1-Alpha-2 (*Man1a2*) Mutants

**DOI:** 10.3389/fphys.2021.658518

**Published:** 2021-07-14

**Authors:** Mylarappa Ningappa, Morayooluwa Adenuga, Kim A. Ngo, Nada Mohamed, Tejaswini Narayanan, Krishna Prasadan, Chethan Ashokkumar, Jishnu Das, Lori Schmitt, Hannah Hartman, Anuradha Sehrawat, Claudia M. Salgado, Miguel Reyes-Mugica, George K. Gittes, Cecilia W. Lo, Shankar Subramaniam, Rakesh Sindhi

**Affiliations:** ^1^Hillman Center for Pediatric Transplantation, Children’s Hospital of Pittsburgh of University of Pittsburgh Medical Center (UPMC), Pittsburgh, PA, United States; ^2^Department of Bioengineering, University of California, San Diego, San Diego, La Jolla, CA, United States; ^3^Department of Cellular and Molecular Medicine, University of California, San Diego, San Diego, La Jolla, CA, United States; ^4^Department of Computer Science and Engineering, and Nanoengineering, University of California, San Diego, San Diego, La Jolla, CA, United States; ^5^Division of Pediatric General and Thoracic Surgery, UPMC Children’s Hospital of Pittsburgh, Pittsburgh, PA, United States; ^6^Rangos Research Center Animal Imaging Core, UPMC Children’s Hospital of Pittsburgh, Pittsburgh, PA, United States; ^7^Departments of Immunology and Computational and Systems Biology, University of Pittsburgh, Pittsburgh, PA, United States; ^8^Histology Core Laboratory Manager, UPMC Children’s Hospital of Pittsburgh, Pittsburgh, PA, United States; ^9^Division of Pediatric Pathology, UPMC Children’s Hospital of Pittsburgh, University of Pittsburgh School of Medicine, Pittsburgh, PA, United States; ^10^Division of Pediatric Pathology, UPMC Children’s Hospital of Pittsburgh, Pittsburgh, PA, United States; ^11^Surgeon-in-Chief Emeritus, UPMC Children’s Hospital of Pittsburgh, Pittsburgh, PA, United States; ^12^Department of Developmental Biology, University of Pittsburgh, Pittsburgh, PA, United States

**Keywords:** congenital lung and liver, multisystem disease, prevention, NAC (N-acetylcysteine), *Man1a2* mutants

## Abstract

**Background:**

Ciliary defects cause heterogenous phenotypes related to mutation burden which lead to impaired development. A previously reported homozygous deletion in the *Man1a2* gene causes lethal respiratory failure in newborn pups and decreased lung ciliation compared with wild type (WT) pups. The effects of heterozygous mutation, and the potential for rescue are not known.

**Purpose:**

We hypothesized that survival and lung ciliation, (a) would decrease progressively in *Man1a2*^+/−^ heterozygous and *Man1a2*^–/–^ null newborn pups compared with WT, and (b) could be enhanced by gestational treatment with N-Acetyl-cysteine (NAC), an antioxidant.

**Methods:**

*Man1a*2^+/–^ adult mice were fed NAC or placebo from a week before breeding through gestation. Survival of newborn pups was monitored for 24 h. Lungs, liver and tails were harvested for morphology, genotyping, and transcriptional profiling.

**Results:**

Survival (*p* = 0.0001, Kaplan-Meier) and percent lung ciliation (*p* = 0.0001, ANOVA) measured by frequency of Arl13b^+^ respiratory epithelial cells decreased progressively, as hypothesized. Compared with placebo, gestational NAC treatment enhanced (a) lung ciliation in pups with each genotype, (b) survival in heterozygous pups (*p* = 0.017) but not in WT or null pups. Whole transcriptome of lung but not liver demonstrated patterns of up- and down-regulated genes that were identical in living heterozygous and WT pups, and completely opposite to those in dead heterozygous and null pups. Systems biology analysis enabled reconstruction of protein interaction networks that yielded functionally relevant modules and their interactions. In these networks, the mutant Man1a2 enzyme contributes to abnormal synthesis of proteins essential for lung development. The associated unfolded protein, hypoxic and oxidative stress responses can be mitigated with NAC. Comparisons with the developing human fetal lung transcriptome show that NAC likely restores normal vascular and epithelial tube morphogenesis in *Man1a2* mutant mice.

**Conclusion:**

Survival and lung ciliation in the *Man1a2* mutant mouse, and its improvement with N-Acetyl cysteine is genotype-dependent. NAC-mediated rescue depends on the central role for oxidative and hypoxic stress in regulating ciliary function and organogenesis during development.

## Introduction

Cilia formation and function are regulated by distinct genes in key developmental pathways and gene networks (McClintock et al., 2008; Van Dam et al., 2013; Toriyama et al., 2016). Dysregulation of these genes underlies a variety of congenital defects. Thus, modeling rescue of ciliary dysgenesis is essential to developing therapeutic approaches. This task can be challenging for diseases with a heterogeneous phenotype that may involve the combined effect of several mutations on multiple organ systems. Primary ciliary dyskinesias (PCD), a heterogenous disease that affects the lungs, sinuses, and middle ear due to abnormal motile cilia illustrates these challenges (Horani et al., 2016). Although autosomal recessive inheritance of causal variants in over 30 genes occurs in over half of all patients, the disease is unexplained in the remainder. Possible reasons include compound heterozygosity or the combined effect of variants in a single allele of two or more ciliary genes (Horani et al., 2016; Poprzeczko et al., 2019). Another example is biliary atresia (BA), a congenital liver disease with a heterogenous phenotype which can also involve cardiovascular and genitourinary systems. BA also has a complex genetic basis (Schwarz et al., 2013) and is associated with sequence variants in the ciliogenesis and planar polarity effector or the CPLANE network genes, *GPC1, ADD3, ARF6, EFEMP1*, and *MAN1A2*. These variants have modest contributions to disease in genome-wide association studies (Cheng et al., 2013; Cui et al., 2013; Ningappa et al., 2015; Chen et al., 2018; So et al., 2020). Thus, model systems for ciliary disease must incorporate the ability to study the effect of mutation burden on the phenotype and its rescue with novel strategies.

Recently, we have shown that *Man1a2*^−/−^ newborn pups which are known to develop lethal respiratory failure due to homozygous deletion of the second *Man1a2* exon, also showed poor lung ciliation, and abnormal biliary epithelial proliferation (Tremblay et al., 2007; So et al., 2020). Further, several ciliary genes and pathways were dysregulated in the liver transcriptome of *Man1a2* null compared with wild type (WT) newborn pups. The affected pathways included hedgehog, epidermal growth factor and transforming growth factor signaling (Anvarian et al., 2019). These pathways have a significant role in branching morphogenesis of epithelial duct networks during the development of organs like the lung, mammary gland, prostate and liver, as well as left-right patterning (Anvarian et al., 2019). Ciliation was difficult to demonstrate in liver of the *Man1a2*^–/–^ mutant mice but was visualized in respiratory epithelial cells with immunostaining of ADP Ribosylation Factor Like GTPase 13B (Arl13b), a regulatory GTPase highly enriched in cilia (Larkins et al., 2011; So et al., 2020). It is not known whether ciliation and survival were adversely affected to an intermediate level by a single copy of the heterozygous deletion in *Man1a2*^+/–^ pups, compared with WT or null pups, and whether these defects could be reversed with treatment.

In this study, we examined whether survival and ciliation was affected adversely by increasing doses of the *Man1a2* exon 2 deletion, and whether these defects could be rescued with an antioxidant like N-acetyl cysteine (NAC). NAC reverses oxidative stress by restoring glutathione stores (Kerksick and Willoughby, 2005). NAC improves ciliation of respiratory epithelial cells infected with respiratory syncytial virus and increases ciliary beat frequency of sinonasal cilia (Mata et al., 2012; Kapusz Gencer, 2016). NAC also prevents congenital heart defects induced by pre-gestational diabetes in animal models (Moazzen et al., 2014). In murine renal epithelial cells, reduced ciliary length induced by oxidative stress is rescued with antioxidants (Kim et al., 2013).

## Materials and Methods

Mannosidase-1- α -2 mice were obtained from Jackson Labs (Catalog#007672) and the colony was maintained by breeding 12 to 48 week old heterozygous *Man1a2*^+/–^ mice. Both male and female mice were fed with NAC solution to provide NAC up to 1 g/kg body weight (Marian et al., 2006) or with the placebo (water), 1 week prior to breeding and continued until delivery of offspring. The survival of newborn pups was monitored every 8 h for 24 h. Surviving pups were euthanized 24 h after birth. Lung and liver tissue were harvested immediately upon death or 24 h after birth. Tissue histology was assessed with hematoxylin-eosin staining. Additionally, the lung tissue was immunostained for the ciliary protein Arl13b (Larkins et al., 2011). The frequency of Arl13b^+^ cells was used as a measure of lung ciliation. All newborn pups were genotyped for *Man1a2* mutation from tail DNA. Detailed methods are provided in the Supplementary Material.

### Immunohistochemistry and Quantification

Lung samples were fixed overnight with 10% Neutral buffered formalin (Leica Biosystems Inc., Buffalo Grove, IL, United States), dehydrated using 30% sucrose overnight then embedded in optimal cutting temperature (OCT) compound, snap-frozen by liquid nitrogen, and sectioned at 7 μM. Heat mediated antigen retrieval was performed for 20 min in a steamer using Citrate Buffer (Abcam, Cambridge, MA, United States). Slides were incubated with mouse monoclonal anti-Arl13b antibody (75–287, clone N295B/66, UC Davis/NIH NeuroMab Facility, Davis, CA, United States) at 4°C overnight and then incubated with Cy3-rabbit anti-mouse antibody (Jackson ImmunoResearch Labs, West Grove, PA, United States) for 1 h at room temperature (RT), the following day. Nuclear staining and mounting were performed using Fluoroshield with DAPI (Sigma-Aldrich, St. Louis, MO, United States). The percent ciliation was expressed as percentage of Arl13b^+^ cells per section were quantified using ImageJ software from at least six sections that were 100 μm apart for each mouse offspring. This measurement was expressed as percent ciliation.

#### Quantitative Real Time PCR

Total RNA was extracted from lung and liver of mice offspring using RNeasy Mini Kit (Qiagen, Valencia, CA, United States) and reverse-transcribed to cDNA using High-Capacity cDNA Reverse Transcription Kit (Thermo Fisher Scientific, Waltham, MA, United States). Quantitative PCR was performed with SYBR Green/ROX qPCR master mix (Fermentas, Glen Burnie, MD, United States) using primers for *Man1a2* exon-2 (forward: 5′-CAAAGTAGCCCAAGCAATGAA 3′, reverse:5′-TCTTGAT CTCCATGTCTTCTGG-3′), *Arl13b* (forward: 5′-ACCAGTGGT CTGGCTGAGATTG-3′, reverse:5′-CATCACTGTCCTTCTCC ACGGT-3′), *Ift74* (forward: 5′-GGCACAGATAGAAGCCAGC ATC-3′), reverse:5′-CAGCTCTTGGTTGGTGATGGAG-3′), and *Dnah11* (forward: 5′-GCCATCACTTCGGTTCCAGAGA-3′, reverse:5′-GGTCTCTTTAGGACTGTCAGGAG-3′), and beta-actin (forward: 5′-ctaaggccaaccgtgaaaag-3′, reverse: 5′-accagaggcatacagggaca-3′) (IDT DNA technologies, Coralville, IA, United States). Target gene expression levels were normalized to beta-actin.

### Lung Morphology

We performed hematoxylin-eosin staining of lung sections to assess morphology in placebo and NAC-treated pups in live WT and heterozygous pups, and dead heterozygous and null pups.

#### Whole Transcriptome RNA Sequencing

Total RNA samples from explanted lung and liver tissue from each of NAC and Placebo groups were pooled from 3 independent experiments per pool for live WT, live *Man1a2*^+/−^, dead *Man1a2*^−/−^, and from 2 independent experiments for dead *Man1a2*^+/−^. The RNA quality evaluated by the 5300 Analytical Fragment Analyzer (Agilent, Santa Clara, CA, United States) showed an average RIN value of 9.25 with a range of 7.9–10 (Supplementary Table 1). RNA libraries were generated using Illumina Truseq stranded mRNA. Purified RNA was fragmented using divalent cations under elevated temperature, and fragments copied into first strand cDNA using reverse transcriptase and random primers, followed by second strand cDNA synthesis using DNA Polymerase I and RNase H. After adding a single “A” base, cDNA was ligated to the adapter, purified, and enriched with PCR to create the final cDNA library. Libraries were validated using KAPA Biosystems primer premix kit with Illumina-compatible DNA primers and Qubit 2.0 fluorometer. Quality was examined using Agilent Bioanalyzer TapeStation 2200, and libraries pooled at a final concentration 1.8 pM. Cluster generation and 100 bp paired-end sequencing was performed on Illumina Novaseq 6000 to obtain up to 20 million reads.

mRNAseq data were processed by first trimming adapters. Trimmed reads were then aligned to Ensembl mouse reference genome (version Mouse.B38) using the Omicsoft Sequence Aligner (OSA) (Hu et al., 2012) on Array Studio V10.1, with index built to Ensembl genome for cDNA and ncRNA genome sequences. Uniquely mapped and quantified transcript abundances from OSA output were imported into R software (version 4.0.3) for downstream differential analysis. Differential expression analysis of mRNAseq data was performed using edgeR package (version 3.30.3) (Robinson et al., 2010; McCarthy et al., 2012) in R software. Differentially regulated genes in NAC-treated offspring were identified using the two criteria: under the exact test with dispersion set to biological coefficient of variation (bcv) to 0.2 method and |log_2_ fold change (FC)|≥1 compared to placebo offspring. Furthermore, the expression values of identified differentially expressed transcripts were visualized in log_2_FC of FPKM using the heatmap package (version 1.0.12) in R without any clustering.

#### Gene Enrichment Analysis of Significant Differentially Expressed Genes in NAC-Treated Newborn Pups

Gene ontology analysis for biological processes indicated by differentially regulated genes in NAC-treated mice was performed using enrichR package (version 2.1) in R software. Also, with enrichR package, functional enrichment for KEGG 2019 mouse pathways was used to assess for enrichment of significant biological pathways using KEGG 2019 mouse database. Significant GO terms for biological processes and KEGG pathways were selected under the adjusted *p*-value cutoff of 0.05 (Chen et al., 2013; Kuleshov et al., 2016). Important biological processes and pathways related to NAC effects on lung morphology, developmental pathways and significant processes were further analyzed in protein-protein interaction network.

Differentially expressed genes were identified in (a) the CPLANE network of 2436 unique genes (Toriyama et al., 2016) and the related PCD ciliopathies (Bergström et al., 2012), (b) oxidative stress response genes (Rotblat et al., 2013; Kim et al., 2018) and GO:0034599, related cellular response to hypoxia (GO:0071456), and glutathione metabolic processes, GO:0006749, (c) lung organogenesis pathways of lung development (GO:0030324 and GO:0060428), branching morphogenesis of an epithelial tube (GO:0048754), and endothelial tube morphogenesis (GO:0061154 and GO:0001885). (d) To understand the role of the genotypic background, we also evaluated differentially expressed genes among interactors with Man1a2 in the STRING database, the N-glycan synthesis (KEGG pathway_hsa00510) and the unfolded protein response (GO:0006986; Reactome pathways_R-HSA-381119) pathway, which mediate the endoplasmic reticulum stress response. Man1a2 regulates proteoglycan maturation within the Golgi complex.

#### Integrative Network Reconstruction

The mouse DEG-encoded proteins in NAC-treated offspring and protein-protein interaction (PPI) networks were generated using online STRING^1^ with a high confidence interaction score of 0.900 and active interaction sources from databases and experiments. The protein interaction relationship network was then imported into Cytoscape (version 3.8.2) by stringApp (version 1.6.2) for visualization (Shannon et al., 2003; Chin et al., 2014; Doncheva et al., 2019).

### Statistical Methods

Statistical tests were performed using GraphPad prism software and included Fisher’s exact test, Kaplan-Meier survival analysis using the log-rank (Mentel-Cox) test, ANOVA test for one-way analysis of variance between three groups, and the Holm-Sidak’s test for pairwise comparisons.

## Results

### Man1a2 Genotype Determines Survival, and Lung Ciliation in Newborn Pups

**FIGURE 1:**
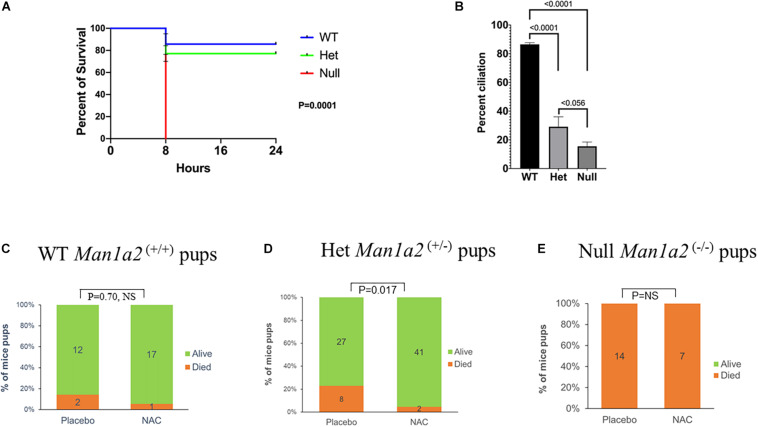
Kaplan-Meier survival curves **(A)** and lung ciliation **(B)** in newborn pups by genotype, WT, heterozygous (*Man1a2*^+/–^) and null (*Man1a2*^–/–^). Effects of placebo and NAC exposure on survival in WT **(C)** and heterozygous **(D)** newborn pups, and null **(E)** dead pups. The percent ciliation was expressed as percentage of Arl13b^+^ cells quantified using ImageJ software.

Survival analysis showed a dependency on the number of copies of the *Man1a2* deletion. The survival of newborn pups decreased, from 85% in WT to 76% in heterozygous, and no survival in homozygous *Man1a2* mutants, *p* = 0.0001, Log rank test (Figure 1A). This was accompanied by a corresponding genotype-dependent decrease in the frequency of Arl13b^+^ respiratory epithelial cells in the lungs, from a mean of 83% in WT to 27% in heterozygous, and 16% in null pups, *p* = 0.0001, ANOVA (Figure 1B).

### Gestational Treatment With NAC Improves Survival of*Man1a2*^+/–^ Heterozygous Pups

Next, we evaluated whether NAC could rescue newborn one-day old *Man1a2* mutant pups. Gestational treatment with NAC resulted in survival of 41 of 43 or 95% of *Man1a2*^+/–^ heterozygous pups compared with 27 of 35 or 77% placebo-treated heterozygous pups (*P* = 0.017) (Figure 1D). NAC treatment of wild-type pups did not show significant survival difference when compared to placebo-treated WT pups, 94 vs. 86% (*p* = 0.70, NS) (Figure 1C). All *Man1a2*^−/−^ pups in the NAC supplemented and placebo groups died within 1 h of birth (*P* = NS) (Figure 1E).

### Gestational NAC Treatment Improves Lung Ciliation and Ciliary Gene Expression in Heterozygous *Man1a2*^+/–^ Pups

**FIGURE 2:**
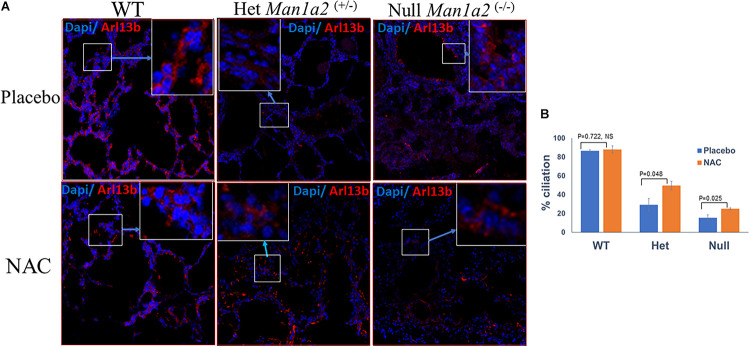
**(A)** Confocal microscopy show Arl13b^+^ cells and nuclei (blue, Dapi) in the lung sections of placebo (tow row) and N-Acetyl-cysteine-treated *Man1a2* WT and heterozygous (*Man1a2*^+/–^) newborn pups, and null (*Man1a2*^–/–^) dead pups. Scale bars: 50 μm. **(B)** Mean ± SEM frequencies of Arl13b^+^ cells in lungs from the three groups of pups. The percent ciliation was expressed as percentage of Arl13b^+^ cells quantified using ImageJ software. WT, Wild type; Het, Heterozygous *Man1a2*^(+/–)^; Null, *Man1a2*^(–/–)^. The inset shows a high-magnification image of the Arl13b^+^ cells area represented by the white rectangle. Four replicates were performed for all experiments. Error bars: SEM.

Heterozygous *Man1a2*^+/–^ pups from NAC-treated group exhibited significantly higher percent lung ciliation or frequencies of Arl13b^+^ cells, 49.7 vs. 29.1% (*P* = 0.048) and expression of the corresponding Arl13b gene (*p* = 0.002) in the lung compared with heterozygous pups in the placebo-treated group (Figure 2, Supplementary Table 2). Similarly, lung tissue from NAC-supplemented *Man1a2*^–/–^ null pups exhibited a significantly higher percent lung ciliation than the placebo-treated group (25 vs. 15.5%, *p* = 0.025); however, the numerically higher expression of the corresponding Arl13b gene did not achieve statistical significance (*p* = 0.081) (Supplementary Table 2). In WT *Man1a2* pups treated with NAC, percent ciliation was unaffected despite higher *Arl13b* gene expression (*p* = 0.044), compared with placebo (Supplementary Table 2).

We have previously shown downregulation of the ciliary genes, *Ift74*, and *Dnah11* in the liver, and of the protein Arl13b in the lungs of untreated *Man1a2*^–/–^ mutants (So et al., 2020). Here we found that NAC exposure resulted in upregulation of all three genes in lung tissue from newborn pups of all genotypes (Supplementary Table 2). Surprisingly, NAC exposure also enhanced expression of the *Man1a2* exon-2 transcripts in heterozygous and WT newborn pups. We did not detect *Man1a2* exon-2 transcripts in lungs from *Man1a2*^–/–^ null newborn pups in NAC-treated and placebo groups, because these pups lacked the corresponding DNA sequence. This experiment shows that NAC exposure enhanced ciliogenesis in the lung. Further, oxidative stress and its modulation by NAC also appears to regulate *Man1a2* expression.

### The Effects of N-Acetyl Cysteine on Lung Morphology and Developmental Pathways Are *Man1a2* Genotype Dependent and Organ-Specific

**FIGURE 3:**
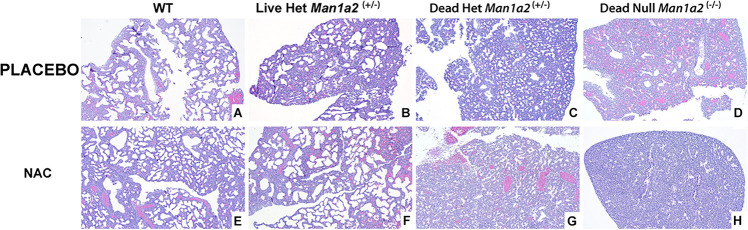
Lung morphology showing striking differences in alveolar development in mice treated with placebo (upper row **A–D**) or NAC (Lower row **E–H**). **(A)** Wild type-placebo; **(B)** Heterozygous, live-placebo; **(C)** Heterozygous, dead-placebo; **(D)** Null, dead-placebo; **(E)** Wild type-NAC; **(F)** Heterozygous, live-NAC; **(G)** Heterozygous, dead-NAC; **(H)** Null, dead-NAC Upper row shows animals treated with placebo. There is a progressive arrest in alveolar development WT to dead null pups. NAC treatment (lower row) has a similar progressive arrest in alveolar development. However, live NAC-treated heterozygous pups show higher counts and size of alveoli similar to NAC-treated WT pups. WT, Wild type; Het, Heterozygous *Man1a2*^(+/–)^; Null, *Man1a2*^(–/–)^.

The structure of pulmonary alveolus of newborn mice includes an alveolar space of approximately 40 to 80 μm, depending on the sectioning level of the specimen from which the measurement results are obtained. The alveolar epithelium is still cuboidal at first and matures into simple squamous type a few days after birth. Generally, the interalveolar septa are 2 to 4 cells thick, and are composed of alveolar epithelial cells and interalveolar capillaries (Kaufmann, 1992; Rieger-Fackeldey et al., 2014). In our study, alveolar development differed visibly with larger and more numerous alveoli in living heterozygous and WT pups and fewer and smaller alveoli in null and heterozygous pups that died in the placebo treated groups (Figures 3A–D, Supplementary Figures 1A–D). NAC treatment enhanced the numbers and size of the alveoli in live heterozygous pups (*P* = 0.01), but not in heterozygous and null pups that died (Figures 3E–H and Supplementary Figures 1E–H, 2, and Supplementary Table 3).

**FIGURE 4:**
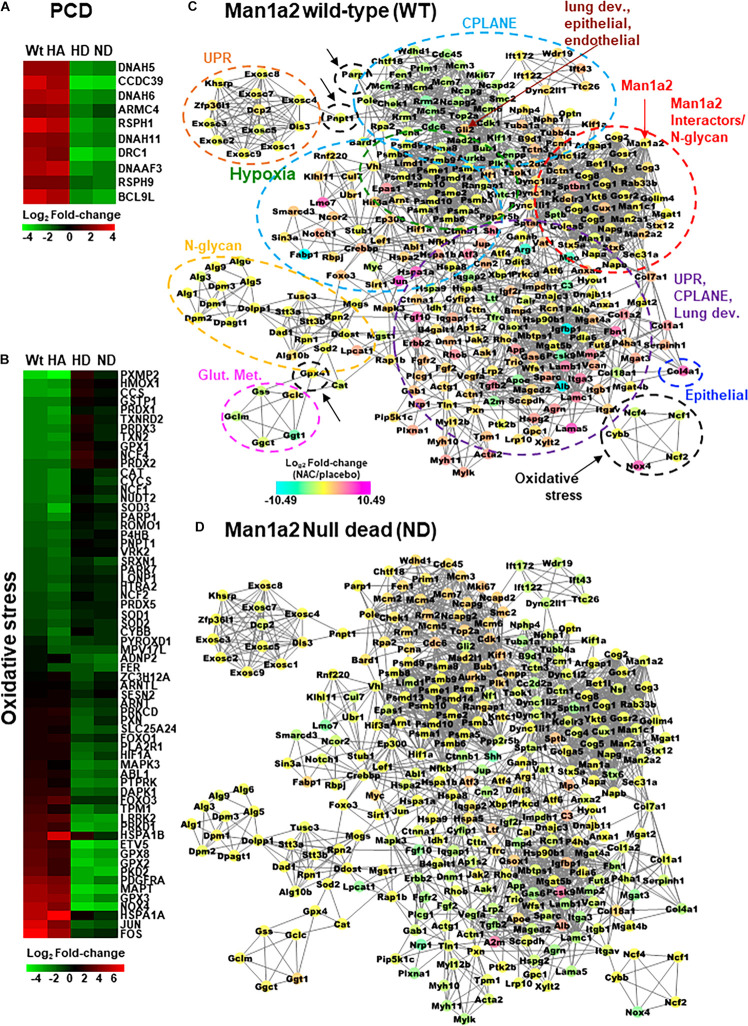
Heatmaps with differentially expressed **(A)** primary ciliary dyskinesia genes and **(B)** selected oxidative stress genes in NAC-treated *Man1a2* pups. Protein-protein interaction (PPI) network with 297 of 847 proteins corresponding to differentially expressed genes in lungs from NAC-treated **(C)** wild type (WT) and **(D)** *Man1a2* null dead (ND) pups. Illustrative heatmaps show differentially expressed **(A)** primary ciliary dyskinesia genes and **(B)** selected oxidative stress genes in NAC-treated pups. Dashed circles in **(C)** identify functional clusters: Man1a2-interactors and N-glycan synthesis (red with arrow), oxidative stress (black with arrows), hypoxia signaling (green), glutathione metabolism (Glut. Met., pink), unfolded protein response (UPR, orange), N-glycan synthesis (yellow), Epithelial tube morphogenesis (blue), and multifunctional cluster (UPR, CPLANE and lung development (purple). Gli2 (maroon arrow) is a key gene in lung epithelial, and endothelial tube development. Multiple clusters circled by light blue dashed lines consist of CPLANE proteins. Node colors indicate the mRNA expression in log_2_ Fold-change: down regulated genes are in cyan, upregulated genes are in magenta and no log2 fold-change genes are in yellow color.

For transcriptomic analysis, we selected only those genes in the abovementioned pathways for which NAC-induced differential expression of log_2_FC could be measured in all four groups. CPLANE and PCD genes were predominantly upregulated in the lungs of live heterozygous and WT offspring and downregulated in lungs from dead heterozygous and null pups (Supplementary Table 4 and Supplementary Figure 3). Examples were the PCD genes *Dnah5, Dnah6, Dnah11, Drc1, Dnaaf3, Armc4, Bcl9l, Rsph1, Rsph9, and Ccdc39* (Figure 4A), and the CPLANE network ciliary genes, *Dync1i2, Dync2li1, Ift43, Ift122, Ift172, and Dnah2.* Consistent with this trend, the majority of genes in lung development, epithelial and endothelial development pathways were also upregulated by NAC treatment in live heterozygous pups compared with those that died (Supplementary Figure 3). Examples of upregulated lung development genes include *Foxf1*, critical for alveolar development, *Foxj1*, a master regulator of ciliogenesis, and *Bmp4*, a member of the transforming growth factor pathway. This predominantly upregulated gene expression pattern in live heterozygous pups was also seen in live WT pups. In contrast, dead null pups and dead heterozygous pups demonstrated predominantly downregulated genes in these pathways (Supplementary Table 4 and Supplementary Figure 3). Corresponding liver tissue from each group showed downregulated genes in these pathways. Upregulation of these genes in liver from dead heterozygous pups was an exception. Lung tissue from live heterozygous and WT pups demonstrated a more even mix of upregulated and downregulated genes in oxidative stress response pathways (Figure 4B, Supplementary Figure 4). For example, oxidative stress response *genes, Nfkb1* and *Hif1a* were upregulated while inflammatory genes *Il1a* and *Il18bp* were downregulated in lungs from live heterozygous and WT offspring. An opposing pattern of expression of these genes was seen in the lung tissue of dead heterozygous and null pups. With the exception of liver from dead heterozygous pups, liver from the other groups largely showed downregulation of the stress response genes (Supplementary Table 4 and Supplementary Figures 3, 4).

We performed western blot analysis to determine whether increased transcript abundance of Foxj1 and Hspa1a (Hsp70) in lung tissue of NAC-treated WT and Het live pups was accompanied by corresponding increase in protein expression. Western blot analysis showed a trend toward enhanced expression of Foxj1 and Hsp70 in the NAC-treated Het pups by 1.52 and 4.35-fold, respectively, when compared with placebo-Het pups, but the data were not significant (Supplementary Figure 5). Similarly, compared with placebo-WT pups, NAC treatment showed a trend toward increased expression of Foxj1 and Hsp70 in WT pups by 1.51 and 1.82-fold, respectively. These differences were not significant (Supplementary Table 4 and Supplementary Figure 5).

#### N-Acetyl Cysteine-Induced Interactions Between Oxidative Stress and Developmental Pathways

**FIGURE 5:**
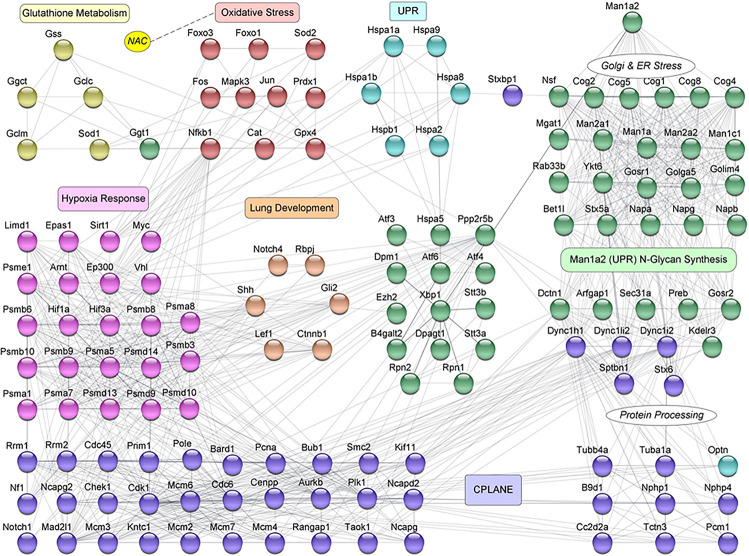
Protein-protein interaction (PPI) network for selected proteins corresponding to 140 differentially expressed genes in NAC-treated newborn pups. This mechanistic network represents the essence of regulatory crosstalk between the key genes that belong to the different functions of interest. The nodes are color coded to represent the different functional mechanisms that are involved in the process of Lung Development. They are represented as: Man1a2 N-Glycan Synthesis (green), Unfolded Protein Response (UPR, light blue), Oxidative Stress (maroon), Glutathione Metabolism (olive green), Hypoxia Response (pink), Lung Development (orange brown), and CPLANE (purple). Genes in Man1a2 N-Glycan Synthesis, that are involved in Golgi and Endoplasmic Reticulum stress, and protein processing are marked separately, and their interactions with CPLANE genes are evident. The influence of NAC (yellow) on the Oxidative Stress pathway genes is represented by a dotted line.

Using the STRING database, we constructed a murine network of experimentally validated PPI with confidence levels of 0.9 for 847 differentially expressed genes (Supplementary Table 4) in the abovementioned pathways. The network consisted of 297 nodes or proteins, each with 4 or more interactions, for a total of 2124 total interactions or edges (Figures 4C,D and Supplementary Figure 6). The 297 proteins within the PPI network included 138 from CPLANE, 4 from endothelial, 4 from epithelial, 4 from lung development, 26 from Hypoxia, 4 from glutathione metabolism, 31 oxidative stress, 3 UPR, and 83 Man1a2-interacting proteins (Supplementary Table 5). Networks were constructed for lung transcripts from WT, live heterozygous, dead heterozygous and dead null pups (Figures 4C,D, Supplementary Figures 6A,B). In each network, a central subnetwork of proteasomal hypoxia signaling proteins, e.g., PSMA7, demonstrated extensive interactions with other proteins in oxidative stress, e.g., Nfkb1, ciliary, e.g., Top2a, lung development, Ctnnb1, or other pathways. These networks reflect opposing trends for gene regulation in live compared with dead pups. Additional analysis in R revealed a single subnetwork or cluster consisting of 75 proteins which illustrates the extensive crosstalk between all key pathways evaluated in this study (Figure 5, Supplementary Table 6). These 75 proteins consist of 37 proteins in the stress response pathways of hypoxia signaling-23, unfolded protein response-8, and oxidative stress-6, and 37 genes in developmental pathways, CPLANE-31, epithelial tube morphogenesis-4, and endothelial tube morphogenesis-2 (Supplementary Figure 7). Twenty-eight differentially expressed transcription factors (TFs) among the 297 proteins within the PPI network further illustrate this cross-talk by their participation in oxidative stress-8, hypoxia signaling-3, Man1a2-interacting pathways-5, CPLANE network-6, endothelial-2 and epithelial morphogenesis-2, and lung development-2 (Supplementary Figures 3, 4 and Supplementary Table 7). To further understand how NAC may rescue lung development in *Man1a2* mutants, we added proteins corresponding to 65 additional DE genes from the lung transcriptome to this 75-protein cluster. These additional genes participate in glutathione metabolism, a potential target of NAC, and N-glycan synthesis, a target of the Man1a2 enzyme. The resulting functional network of 140 genes demonstrated extensive crosstalk between several functional modules (Figure 5). This cross-talk suggests that in *Man1a2* mutants, abnormal synthesis and glycosylation affects CPLANE network and lung development pathway proteins. Members of these developmental pathways such as Shh in hedgehog, Notch 1 and Notch 4 in Notch, and Ctnnb1 in Wnt likely recruit the unfolded protein response, hypoxia signaling, and oxidative stress responses. These stress responses can be mitigated with NAC (Figure 5).

### Comparative Gene Ontology Enrichment Analysis Identifies Blood Vessel Development and Tube Morphogenesis as a Target of NAC-Mediated Rescue

To confirm whether our findings provide a model for human lung development, we performed a comparative enrichment analysis using WebGestalt (Liao et al., 2019). We evaluated 3223 previously reported genes which reflect common processes enriched in the transcriptome of the developing human fetal lung between days 53–154 (Kho et al., 2010), and 847 genes used in the network (Figures 4C,D and Supplementary Figure 6). Common cellular components enriched in both gene sets were Golgi or endoplasmic reticulum, microtubular cytoskeleton and plasma membrane (Supplementary Table 8). Common biological processes enriched in both gene sets were blood vessel development and tube morphogenesis. The molecular function of oxidative stress was enriched in mouse lung with gestational NAC treatment, but not in the untreated fetal human lung. Thus, blood vessel development and tube morphogenesis are important sites of NAC-induced changes in oxidative stress.

## Discussion

Proper development depends on the effects of gene expression and its variation on phenotypes in an organ-specific manner. Here we explored the effects of heterozygous or homozygous deletion of the second exon of *Man1a2* on lung development and survival. Consistent with the first description of this model, the homozygous *Man1a2* mutation was lethal (Tremblay et al., 2007). Survival and ciliation decreased in a genotype-dependent manner (Figures 1, 2). Newborn pups with the heterozygous mutation developed intermediate defects in survival and lung ciliation as measured by the frequency of Arl13b^+^ respiratory epithelial cells. All homozygous *Man1a2* mutants died. Gestational treatment of heterozygous parents with NAC improved lung ciliation and survival of heterozygous pups. Although NAC treatment also increased lung ciliation in null pups, it did not prevent lethality. One explanation for this discrepancy could be that the increase in ciliation with NAC treatment was insufficient in null pups. Other unknown factors may also be responsible.

These observations are in line with others which show that homozygous mutations can be lethal, while heterozygous mutants survive but develop anomalies (Favor et al., 2009). Homozygous mutations in the paired box protein, *Pax6*, which promotes development of the eye, nose, central nervous system, and the pancreas, are lethal while heterozygous mutations cause aniridia and other defects (Schedl et al., 1996; Yasuda et al., 2002). Homozygous mutations in the transcription factor *Pdx1* cause pancreatic agenesis, but heterozygous mutations lead to manifestations of diabetes (Fujimoto and Polonsky, 2009).

The benefit of NAC treatment on survival likely resulted from augmented ciliogenesis as suggested by upregulation of the ciliary genes, *Arl13b, Dnah11*, and *Ift74* in lungs of NAC-treated newborn pups with each genotype (Supplementary Table 3). We have previously reported reduced expression of these genes or corresponding proteins in *Man1a2* null compared with WT newborn pups (So et al., 2020). Interestingly NAC treatment also enhanced *Man1a2* exon 2 transcription in lungs from heterozygous and WT pups but not in null pups who lacked the exon 2 DNA sequence. Other genes in candidate pathways, which were differentially expressed after NAC treatment in pups of each genotype demonstrated striking differences in expression patterns in surviving and dead pups (Supplementary Figures 3–7). We observed a predominant upregulation of ciliogenesis and lung development pathway genes in heterozygous live pups, and predominantly downregulated genes in dead heterozygous pups. Ciliogenesis pathways were represented by the CPLANE network genes and the related PCD genes. Lung development was represented by epithelial and endothelial tube morphogenesis and lung development pathway genes. These patterns are consistent with the fact that loss of function mutations in the CPLANE and PCD genes contribute to congenital anomalies (Horani et al., 2016; Toriyama et al., 2016) and adequate function of lung development genes is a prerequisite for normal lung development. That these changes contributed to survival or death is further supported by the fact that upregulated genes in live NAC-treated heterozygous pups were also upregulated in live WT. Downregulated genes in dead NAC-treated heterozygous pups were also downregulated in dead NAC-treated null pups.

Analysis of experimentally validated PPI between differentially expressed genes suggested strong interactions between stress response pathways, the ER/Golgi synthetic and stress response machinery, and pathways essential for lung development including ciliogenesis. This is illustrated by the near identical mix of functional modules in the network comprising 297 nodes (Figures 4C,D and Supplementary Figure 6), the 28 transcription factors among the 297 nodes (Supplementary Figure 4). NAC-induced changes in gene expression in key functional modules in WT, dead null, live heterozygous, and dead heterozygous pups (Figures 4C,D and Supplementary Figure 6) are illustrated in greater detail in heatmaps for PCD and selected oxidative stress genes (Figures 4A,B). These interactions are to be expected because the ER and the Golgi complex are sites for the synthesis and maturation of glycoproteins such as tubulin which is present in the microtubules, a component of the cellular cytoskeleton and cilia (Long and Huang, 2020). Stress-induced pathways originating from the ER modulate pericentriolar proteins such as pericentriolar material 1 or Pcm1, which are in proximity to basal bodies, to facilitate ciliogenesis (Chavali and Gergely, 2013). Ciliogenesis is also regulated by the interaction of CPLANE proteins with the basal body protein, JbDS17, to recruit intraflagellar transport (IFT) proteins, which facilitate transport of other proteins to the cilia (Toriyama et al., 2016). The release of IFT proteins from the ER is triggered by diacylglycerol kinase epsilon (Dgke). *Pcm1* and *Dgke* were upregulated in NAC-treated live WT and heterozygous pups and downregulated in dead heterozygous and null pups. Defective glycosylation of ciliary proteins like Jbt17, which is performed by enzymes such as Man1a2 can lead to ciliopathies (Kane et al., 2017).

Our systems biology analysis using 140 of 297 network nodes reveals endotype mechanisms by which the heterozygous *Man1a2* mutation could impair oxidative stress response and lung development pathways (Figure 5). Man1a2 cleaves excess mannose residues during the maturation of N-glycans (Tremblay et al., 2007). This process is regulated in part by the UPR protein, X-box linked protein-1(Xbp1) acting alone or with activating transcription factors 6 and 4 (ATF6 and ATF4) (Dewal et al., 2015). The mutant enzyme can promote accumulation of unfolded or misfolded proteins resulting in Golgi-ER stress and the Xbp1-ATF mediated UPR (Dewal et al., 2015). The UPR can lead to oxidative stress and hypoxic response further potentiated by altered CPLANE and lung developmental proteins, as well as abnormal microtubular architecture which affects organellar morphology. These effects can directly influence lung development mechanisms mediated by sonic hedgehog (*Shh*) Notch (e.g., *Notch1* and *Notch 4*), and Wnt (e.g., *Ctnnb1*) signaling (Supplementary Figure 7). NAC can reverse these changes by replenishing glutathione to counter oxidative stress, upregulating *Hsp70* leading to an efficient UPR (Gleixner et al., 2017), and upregulating *Nfkb1* which promotes alveolarization (Alvira, 2014). This complex interplay between functional pathways provides a framework for further investigations of lung development. Comparative gene ontology enrichment analysis of fetal human lung transcriptomes also confirms the relevance of our model. This comparison strongly implicates blood vessel development and tube morphxogenesis as processes that benefit from NAC-induced changes in oxidative stress in *Man1a2* mutants (Supplementary Table 8). Previous reports support this inference. Lung blood vessel development is rate limiting for epithelial tube morphogenesis (Van Tuyl et al., 2005). NAC promotes vasodilation of coronary vessels via effects on endothelial cells (Andrews et al., 2001), inhibits monocyte adhesion to endothelial cells (Faruqi et al., 1997), and reverses intrauterine growth restriction in guinea pigs by restoring endothelial cell function (Herrera et al., 2017). Thus, systems analysis implicates several pathways which can be interrogated in a follow-up study.

Because ciliation is poorly visualized in the liver, we could not ascertain the effects of NAC on liver ciliation. Nevertheless, analysis of the liver transcriptome from the four subgroups of newborn pups showed a predominant downregulation of most gene sets, or a change opposite to those seen in lung from heterozygous pups. This suggests that the response to oxidative stress and its attempted rescue with a particular agent may be organ specific. This specificity is supported by the clinical experience with NAC. NAC treatment clears lung secretions in several acute and chronic lung conditions (Dekhuijzen and van Beurden, 2006; Li et al., 2018). However, NAC failed to reverse liver failure in a controlled clinical trial (Squires et al., 2013). NAC inhibits liver regeneration and may be hepatotoxic if used for a protracted period (Athuraliya and Jones, 2009).

In conclusion, our findings provide a window into the heterogeneous presentation and possible therapy of several phenotypes with a complex genetic basis. In such phenotypes, risk alleles or mutation(s) impose an additive burden resulting in mild or severe disease. In the case of the *Man1a2* exon 2 deletions, survival and lung ciliation is rescued with NAC in newborn pups with the heterozygous, but not the null genotype. NAC mitigates oxidative and hypoxic stress responses elicited by aberrant structural proteins *in Man1a2* mutants in developing blood vessels and epithelial tubes which drive organogenesis. This rescue is also minimizes the essential role of the unfolded protein response which is essential for the maturation of structural glycoproteins necessary for ciliary and cellular cytoskeleton. Organ-specific developmentally active gene sets and stress response genes will likely dictate whether an alternative antioxidant, or an alternative mechanistic interaction will rescue other congenitally abnormal organ systems with underlying genetic susceptibility.

## Data Availability Statement

All sequencing datasets presented in this study have been deposited to the NCBI Gene Expression Omnibus (GEO) with accession number GSE172035, https://www.ncbi.nlm.nih.gov/geo/query/acc.cgi?acc=GSE172035.

## Ethics Statement

The animal study was reviewed and approved by All mouse experiments were approved by the Animal Research and Care Committee at University of Pittsburgh IACUC (protocol #17051167).

## Author Contributions

MN planned, conducted, interpreted, and described the study results. KP, GG, and CL contributed to study design, interpreted the results, and edited the manuscript. MA, NM, and CA performed the study procedures. HH and AS performed the western blot. MR-M supervised and described morphological evaluation by LS, and alveoli statistical analysis by CS. SS supervised network analysis by KN, TN, and JD interpreted results and wrote the manuscript. RS obtained funding, supervised key aspects of study design, and  wrote the manuscript. All authors contributed to the article and approved the submitted version.

## Conflict of Interest

The authors declare that the research was conducted in the absence of any commercial or financial relationships that could be construed as a potential conflict of interest.

## Footnotes

1 http://string-db.org/

## Abbreviations

ANOVA: Analysis of variance
*Arl13b*: ADP Ribosylation Factor Like GTPase 13B
*Armc4*: Armadillo Repeat Containing 4
BA: Biliary atresia
*Bcl9l*: BCL9 Like
*Ccdc39*: Coiled-Coil Domain Containing 39
cDNA: Complementary DNA
ChEA: Transcription factor regulation inferred from integrating genome-wide ChIP-X
CPLANE: Ciliogenesis and planar polarity effector
Crebbp: CREB Binding Protein
*Ctnnb1*: *C*atenin Beta 1
DNA: Deoxyribonucleic acid
*Dnaaf3*: Dynein Axonemal Assembly Factor 3
*Dnah11*: Dynein Axonemal Heavy Chain 11
*Dnah2*: Dynein Axonemal Heavy Chain 2
*Dnah5*: Dynein Axonemal Heavy Chain 5
*Dnah6*: Dynein Axonemal Heavy Chain 6
*Drc1*: Dynein Regulatory Complex Subunit 1
*Dync1i1*: Dynein Cytoplasmic 2 Light Intermediate Chain 1
*Dync1i2*: Dynein Cytoplasmic 1 Intermediate Chain 2
FC: Fold-change
*Foxf1*: Forkhead Box F1
*Foxj1*: Forkhead Box J1
FPKM: Fragments Per Kilobase of transcript per Million mapped reads
GO: Gene ontology
Het: Heterozygous (*Man1a2*^+/–^)
*Hif1a*: Hypoxia Inducible Factor 1 Subunit Alpha
*Ift122*: Intraflagellar Transport 122
*Ift172*: Intraflagellar Transport 172
*Ift43*: Intraflagellar Transport 43
*Ift74*: Intraflagellar Transport 74
*Il18bp*: Interleukin 18 Binding Protein
*Il1a*: Interleukin 1 Alpha
*Man1a2*: Mannosidase-1- α -2
mRNA: Messenger RNA
NAC: N-acetyl cysteine
ncRNA: Non-coding RNA
*Nfkb1*: Nuclear Factor Kappa B Subunit 1
*Nphp4*: Nephrocystin 4
Null: *Man1a2*^–/–^
OCT: Optimal cutting temperature
PCD: Primary ciliary dyskinesias
PPI: Protein-protein interactions
RNA: Ribonucleic acid
*Rsph1*: Radial Spoke Head Component 1
*Rsph9*: Radial Spoke Head Component 9
*Sptb*: *S*pectrin Beta, Erythrocytic
STRINGdb: Search Tool for the Retrieval of Interacting proteins database
*Stx5a*: Syntaxin 5
*Top2a*: DNA Topoisomerase II Alpha
WT: Wild type.
