# Supplementary figures and images for: Mechanisms of Impaired Lung Development and Ciliation in Mannosidase-1-Alpha-2 (Man1a2) Mutants
Source: Front Physiol. 2021 Jul 14;12:658518. doi: 10.3389/fphys.2021.658518 (PMC8343402; doi:10.3389/fphys.2021.658518)

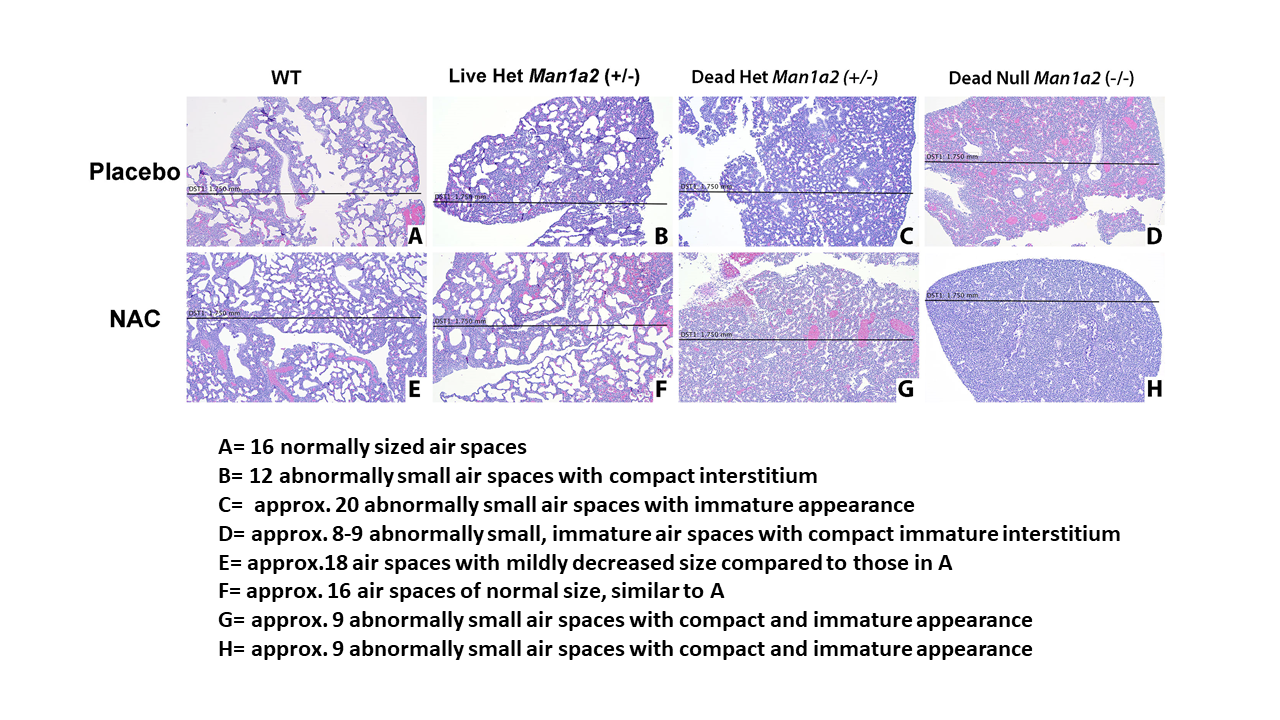

Supplement: Supplementary file 1 [file Image_1.TIF]

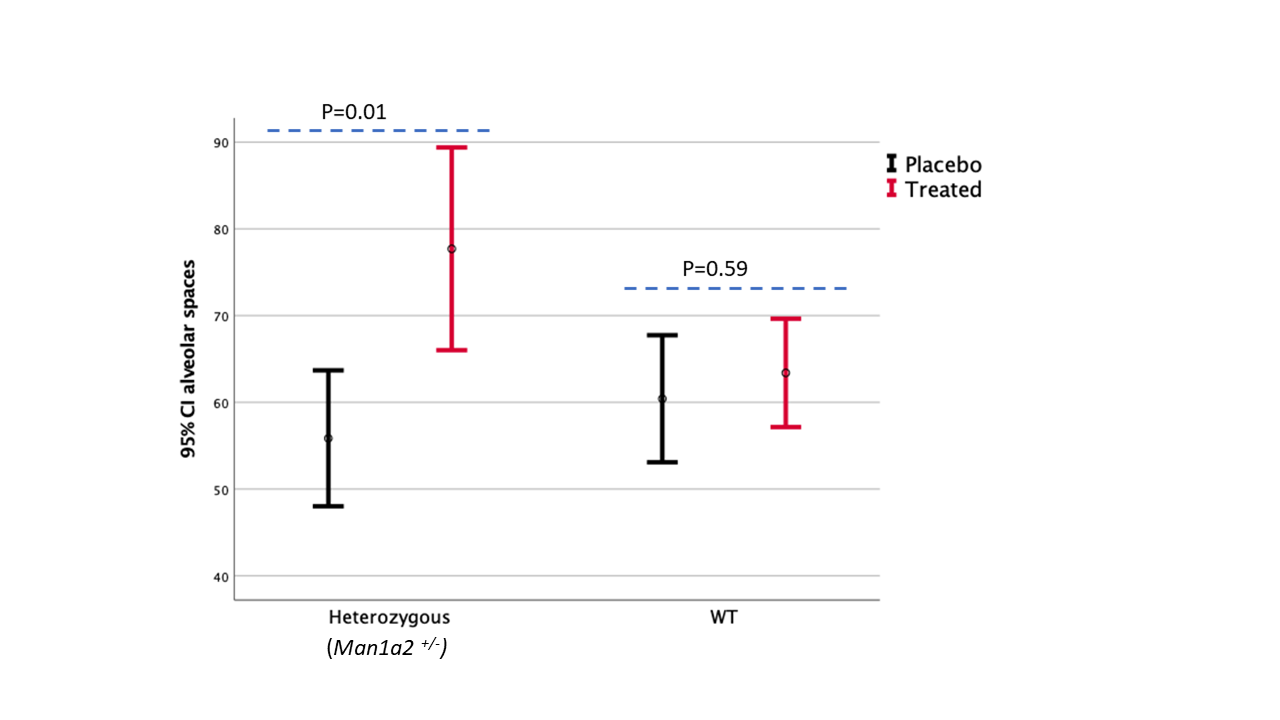

Supplement: Supplementary file 2 [file Image_2.TIF]

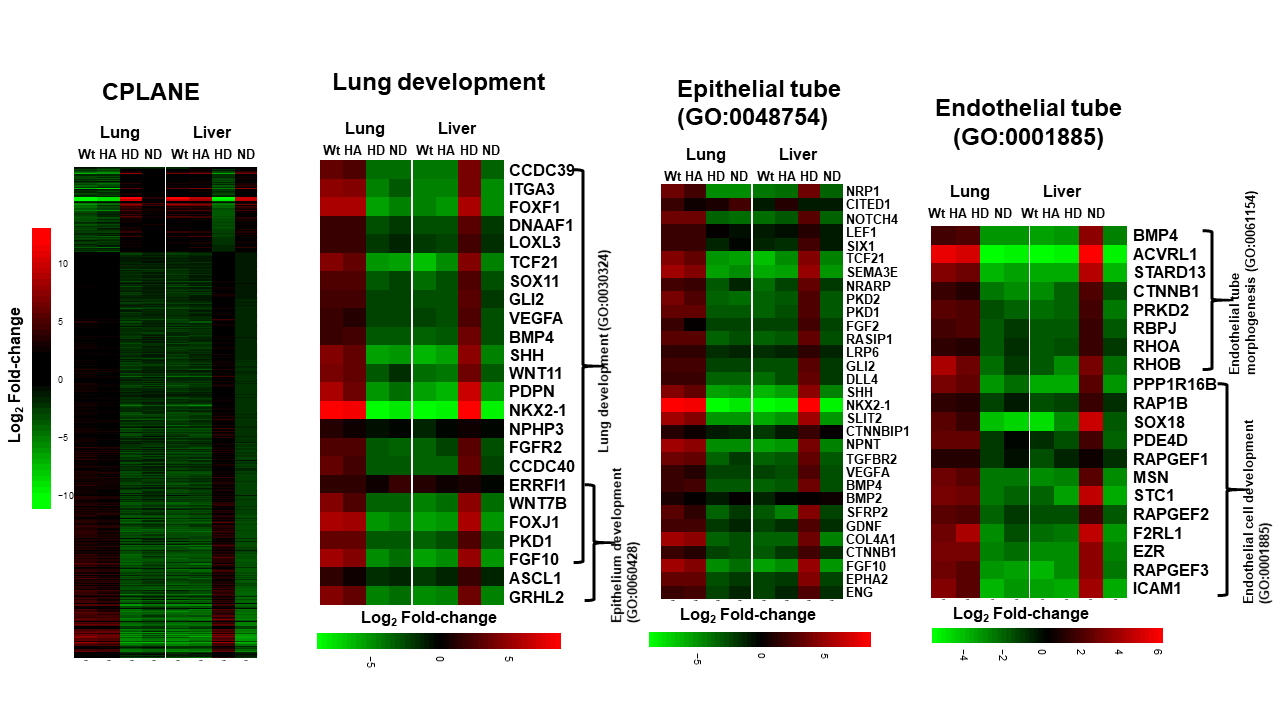

Supplement: Supplementary file 3 [file Image_3.TIF]

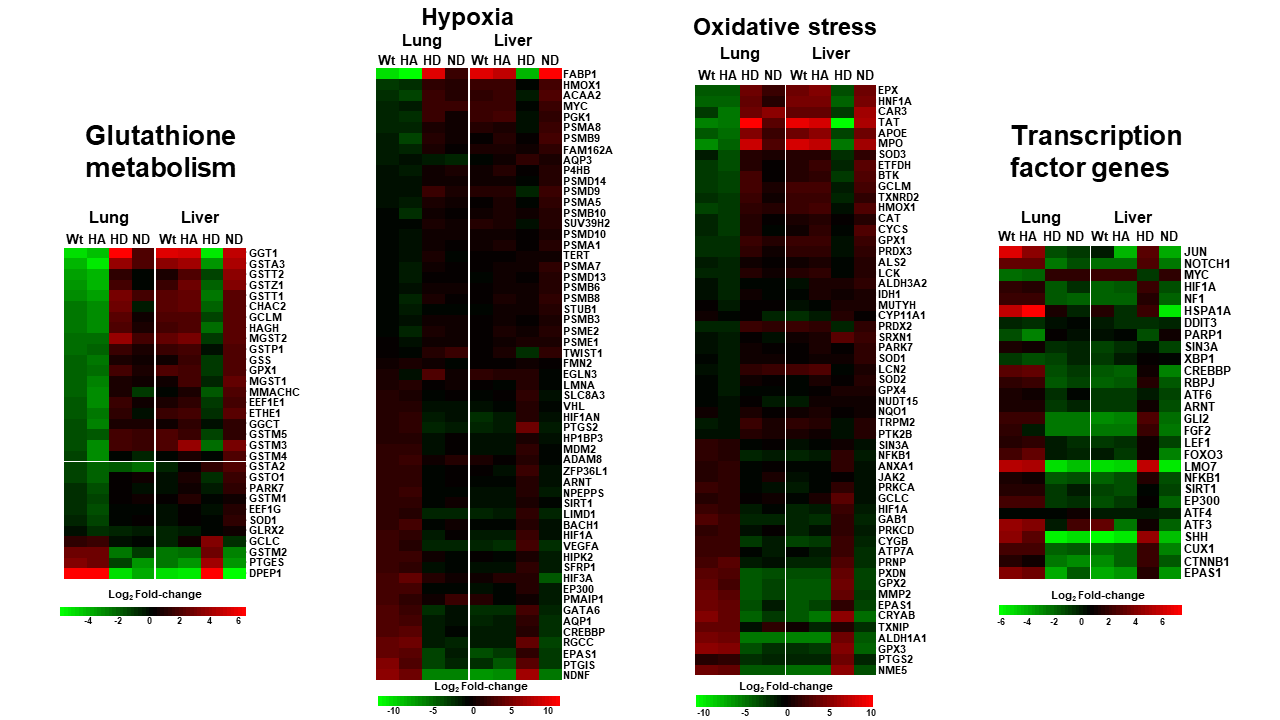

Supplement: Supplementary file 4 [file Image_4.tif]

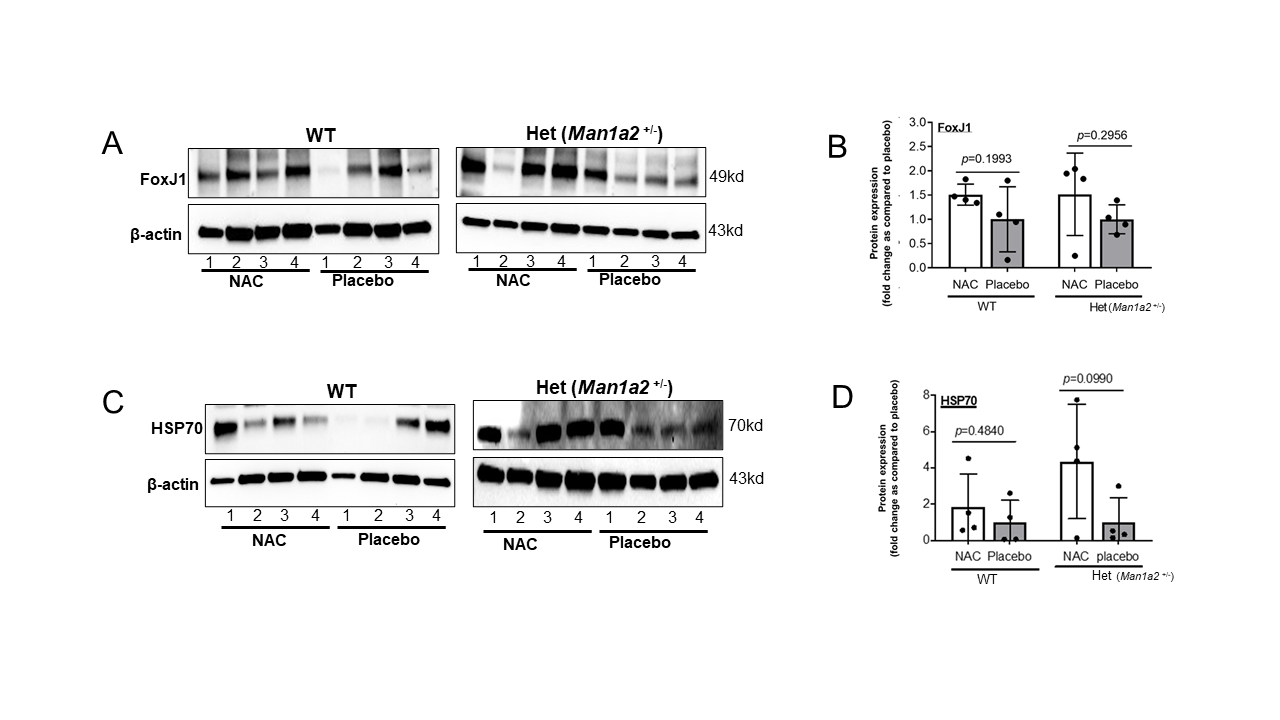

Supplement: Supplementary file 5 [file Image_5.TIF]

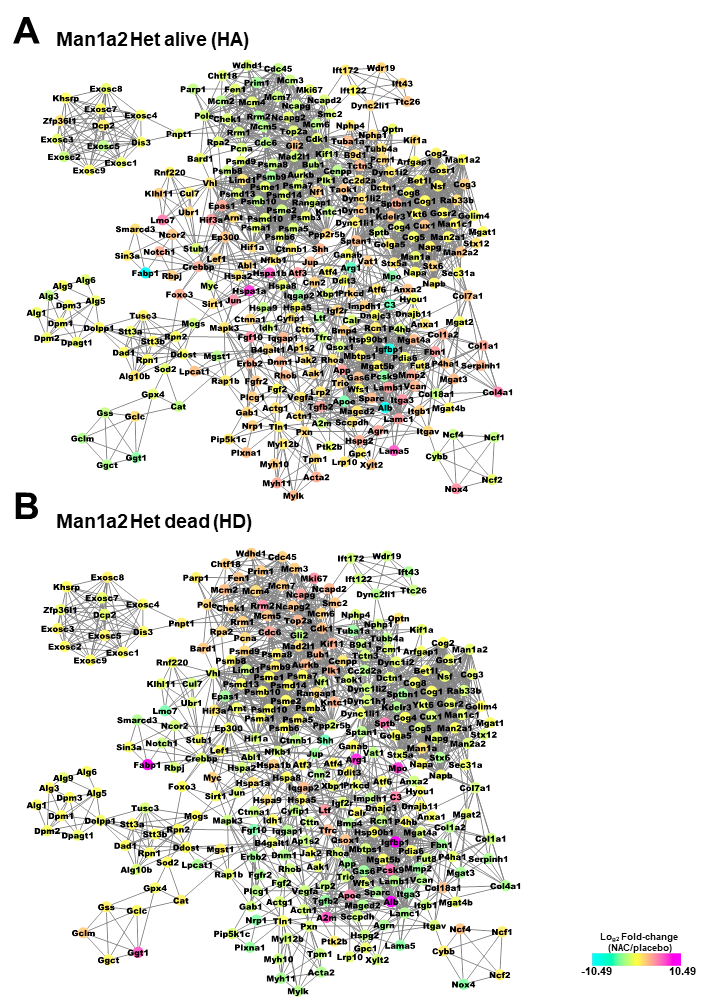

Supplement: Supplementary file 6 [file Image_6.TIF]

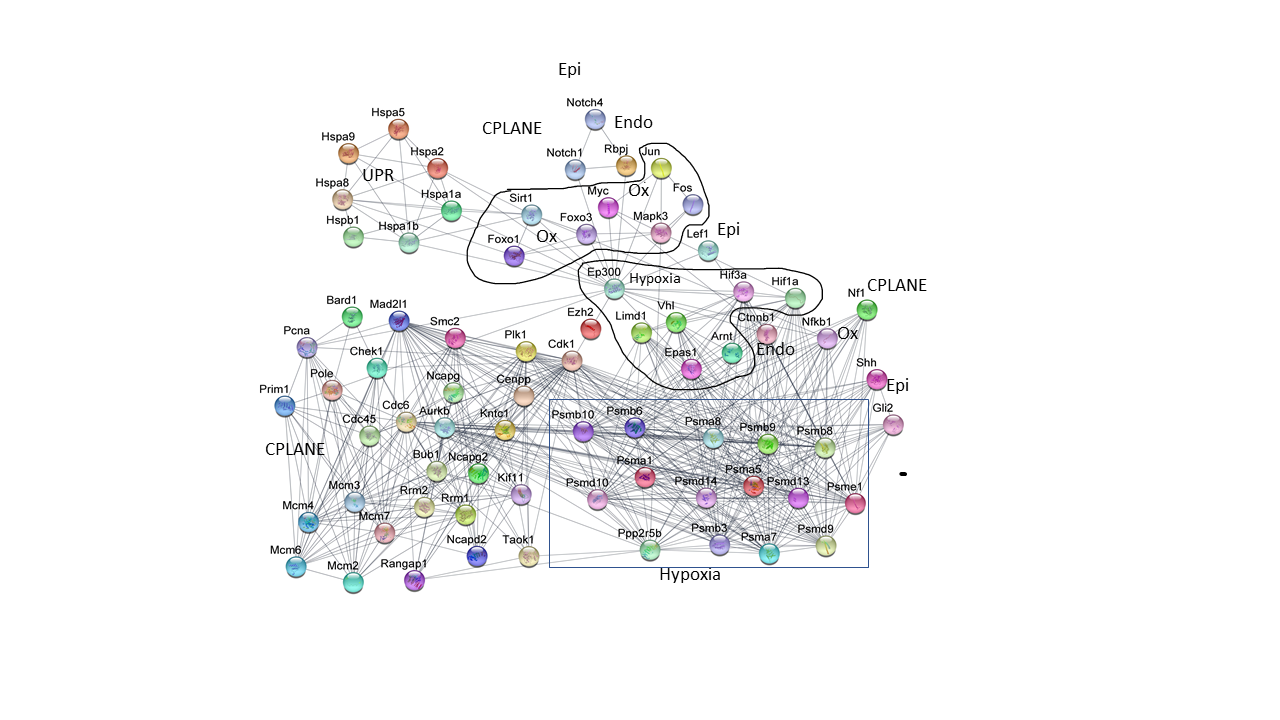

Supplement: Supplementary file 7 [file Image_7.TIF]
