# Supplementary material for: Mechanisms of Impaired Lung Development and Ciliation in Mannosidase-1-Alpha-2 (Man1a2) Mutants
Source: Front Physiol. 2021 Jul 14;12:658518. doi: 10.3389/fphys.2021.658518 (PMC8343402; doi:10.3389/fphys.2021.658518)
Supplement: Supplementary file 9 [file Data_Sheet_2.docx]

**Supplementary materials and methods:**

**Experimental design and N-acetylcysteine (NAC) treatment.** All mouse experiments were approved by the Animal Research and Care Committee at University of Pittsburgh IACUC (protocol #17051167). Man1a2tm1.1Ahe mice (#007672, Jackson laboratory, Chicago, IL) and C57BL/6J (Jackson laboratory, Chicago, IL) were bred under standard conditions with a 12 h light-dark cycle to generate *Man1a2* ^(+/-)^ mice. The *Man1a2* mice ^(+/-)^ with 12- 48 weeks of age were separated in to two groups, placebo, and NAC (N-acetylcysteine). The NAC solution was prepared freshly by dissolving 7g NAC (Sigma-Aldrich, St. Louis, MO) in 800 ml of sterile water to provide NAC up to 1g/kg body weight (Marian et al, 2006). Both male and female mice were fed with the placebo (water) or with NAC solution one week prior to breeding and continued until delivery of offspring. During breeding, female mice were monitored for signs of pregnancy. When pregnancy was identified, the pregnant mouse was isolated into a new cage until delivery of offspring. Once offspring were born, they were monitored for survival at 8-hour intervals up to 24 hours.

**Genotyping.** Genomic DNA was extracted from tail tissue using Kapa mouse genotyping kit (Cape Town, South Africa). Using lysate as DNA template, PCR reactions were performed in GoTaq Green Master Mix (Promega, Madison, WI) with *Man1a2* wildtype primer pair (5'-AAC GTG TAG GGG TCA GTG GT-3') and (5'-GCT CTG CAC AGC ATC TGG TA-3') and the *Man1a2* Mutant primer pair (5'-CAA ATG ACA AAT GGG CTG AG-3') and (5'-GCT CTG CAC AGC ATC TGG TA-3') (IDT, Coralville, IA). PCR was performed at the thermal profile of 94^o^C 3mins for 1 cycle, 94^o^C 1min, 62.5^o^C 30s, 72^o^C 30s for 35 cycles and 72^o^C, 7 mins for 1 cycle. The *Man1a2* WT primer pair produces a fragment of 380bp and the *Man1a2* mutant primer pair produces a fragment of 320bp. The genomic fragments of each mutant and wild type were validated by running on 1.5 % agarose gel.

**Tissue harvesting for histology and gene expression studies.** Newborn pups that died within 24 hours were harvested immediately for liver and lung tissues. Surviving newborn pups were euthanized by decapitation with scissors and dissected through a midline incision extending from the pubic symphysis cranially into the thoracic cavity. This procedure was performed to identify gross abnormalities such as organ anomalies abdominal organ anomalies such as positioning of lung and heart misplacement, and lung hemorrhages. The tail tissue was harvested for genotyping. The lung and liver tissue harvested separately in RNA later for RNA extraction and 10% neutral buffered formalin fixatives (Leica Biosystems Inc., Buffalo Grove, IL) for histological examination, and snap frozen tissue for western blot analysis.

**Western Blotting.** Frozen tissue samples were suspended in NP40 lysis buffer (Thermofisher, Waltham, MA) supplemented with protease/phosphatase inhibitor cocktail (Cell Signaling Technology) and then homogenized via sonication. Homogenates were incubated (on ice, 30 minutes) and centrifuged (12,000 rpm, 4^o^C, 20 minutes) to collect total protein supernatants. Total protein isolated from tissues was quantified by Bicinchoninic Acid Protein assay (QuantiProTM BCA Assay kit, Sigma-Aldrich, St.Louis, MO). Total protein (25սg) was separated on SDS-polyacrylamide gels and then transferred to polyvinylidene difluoride membranes. Membranes were incubated with the following primary antibodies (4^o^C, overnight): rabbit polyclonal anti-FOXJ1 (1:500, Abcam Inc., Boston, MA) and mouse monoclonal anti-HSP70 (1:1000, ProteinTech, Rosemont, IL). Membranes were then incubated in either horseradish peroxidase-conjugated anti-rabbit (1:2000, Jackson ImmunoResearch, West Grove, PA) or anti-mouse (1:2000, Jackson ImmunoResearch, West Grove, PA) secondary antibodies (room temperature, 2 hours). Immunoreactive bands were detected by ultra-sensitive enhanced chemiluminescence (SuperSignalTM Maximum Sensitivity Substrate, ThermoFisher, Waltham, MA). Band quantification was performed utilizing ImageJ software.

**Quantification of lung alveolar spaces.** Hematoxylin-eosin staining was performed on available lung tissue from pups in the NAC treated-live heterozygous (n=2) vs placebo treated-live Heterozygous (n=2), NAC treated-live WT (n=2) vs Placebo treated-live WT (n=2), NAC treated-dead heterozygous (n=1) vs placebo treated-dead heterozygous (n=1), and NAC treated-null (n=1) vs placebo treated-dead null (n=2). The stained slides were scanned with an Aperio CS2 slide scanner (Leica Biosystems, Buffalo Grove, IL, USA) at 40× magnification. The whole slide image at digital zoom up to 40× was visualized using the Aperio ImageScope software. For each case, morphometric analysis of the digital image was performed by a pediatric pathologist. The structure of pulmonary alveolus of newborn mice includes an alveolar space of approximately 40 to 80 µm, depending on the sectioning level of the specimen from which the measurement results are obtained. The alveolar epithelium is still cuboidal at first and matures into simple squamous type a few days after birth. Generally, the interalveolar septa are 2 to 4 cells thick and are composed of alveolar epithelial cells and interalveolar capillaries (Kaufmann, M.H, 1992; Rieger-Fackeldey E et al., 2014). Ten random alveolar spaces were measured in each lung using the Aperio ImageScope software measuring tool. The unpaired t-test was used to compare the mean alveolar size of the total measured values of NAC treated-live heterozygous (n=2) and NAC treated live WT (n=2) with the respective placebo.

**Supplementary Figure Legends:**

**Supplementary Figure 1.**

The photomicrographs taken using a total magnification of 100X to assess the lung development. The intersection air-space counting performed to count the number of alveolar/airway spaces intercepted by the line.  A) placebo WT showed 16 normally sized alveolar and airway spaces and air spaces and E) NAC-treated WT showed approximately 18 air spaces with mildly decreased size compared to those in WT placebo (A). The placebo Het (B) exhibited 12 abnormally small air spaces with compact interstitium. Interestingly, NAC-treated Het (F) restored approximately 16 normal sized air spaces, indicating an increase in alveoli compared to those in placebo Het (B). Dead Het placebo (C) exhibited 20 abnormally small air spaces with immature appearance which is higher than dead Het NAC that showed approximately 9 abnormally small air spaces with compact immature intestitium (G). Both dead null placebo (D) and dead null NAC (H) showed approximately 9 abnormally small air spaces with immature appearance, the lungs are compact, with reduced number of alveolar/airway spaces and a reduced size in them. WT, Wild type; Het, heterozygous (*Man1a2*^(+/-)^); Null, *Man1a2*^(-/-)^.

**Supplementary Figure 2.**

The figure shows mean alveolar size with 95% confidence interval in the lung from NAC-treated live Heterozygous and live WT pups compared with the respective placebo-treated pups. p < 0.05 is considered significant.

**Supplementary Figure 3**. Heatmap of differentially expressed genes for Lung/Structural development including CPLANE network. Heatmaps shows up- (red) and downregulated (green) genes in lung and liver from NAC-treated live WT and heterozygous Man1a2^(+/-)^ pups (HA), and dead heterozygous Man1a2^(+/-)^ (HD) and null Man1a2^(-/-)^ (ND) pups. From left to right heatmaps show genes in ciliary and planar polarity effector (CPLANE) network, lung, epithelial and endothelial tube development pathways.

**Supplementary Figure 4.** Heatmaps shows up- (red) and downregulated (green) genes in lung and liver from NAC-treated live WT and heterozygous Man1a2^(+/-)^ pups (HA), and dead heterozygous Man1a2^(+/-)^ (HD) and null Man1a2^(-/-)^ (ND) pups. From left to right heatmaps show differentially expressed genes in glutathione metabolism, hypoxia signaling, oxidative stress response (additional genes), and also include transcription factors.

**Supplementary Figure 5.** Western blot (WB) shows the protein expression of FoxJ1 (A) and Hspa1a (Hsp70) (C) in the lung tissue of four WT and four *Man1a2* +/- Heterozygous (Het) newborn pups in both NAC-treated and placebo mice. The band quantification was determined using Image J software as described in the methods. The bar graph shows the fold change in protein expression of FoxJ1 (B) and HSP70 (D) compared to placebo. Data represent four independent replicates, mean ± SD.

**Supplementary Figure 6.** Protein-protein interaction (PPI) network with 297 of 847 proteins corresponding to differentially expressed genes in lungs from NAC-treated Man1a2 pups. A) Man1a2 heterozygous alive (HA), and B) Man1a2 heterozygous dead (HD). The functional clusters are indicated in dashed circles labelled in Figure 4C. Node colors indicate the mRNA expression in log_2_ Fold-change: down regulated genes are in cyan, upregulated genes are in magenta and no log2 fold-change genes are in yellow color.

**Supplementary Figure 7.** A key cluster from the network in Figure 4 shows 75 proteins and associated functional modules. Functional modules include CPLANE-cilia planar polarity effector, Ox-oxidative stress, UPR-unfolded protein response, Endo-endothelial tube morphogenesis, Epi-epithelial tube morphogenesis. Additional genes were added to this cluster to create the final network in Figure 5 as described in results.

**Supplementary Table Legends:**

**Supplemental Table 1.** The characteristics of RNA samples isolated from lung and liver tissues of newborn pups with NAC and placebo from 3 independent experiments in each pool from live WT, live Man1a2 +/- (Het), dead Man1a2 -/- (Null) pups, and 2 experiments in each pool from dead Het and dead Null pups. The RNA quality was assessed using Analytical Fragment Analyzer 5300. The RNA quality check of the lung tissues of Het dead and Null dead pups showed that the average RQN number was 8.55 (Range 7.9-9.6) suggesting good quality RNA. Also, all the RNA seq samples including live WT, live Het, dead Het and dead Null pups showed the average RIN number 9.25, with a range of 7.9-10.

**Supplemental Table 2**. Differential expression of selected ciliary genes measured with quantitative reverse transcription-PCR (qRT-PCR) in lungs from NAC-treated WT, heterozygous and null pups.

**Supplementary Table 3.**

Hematoxylin-eosin staining was performed on available lung tissue from pups in the NAC treated-live heterozygous (n=2) vs placebo treated-live Heterozygous (n=2), NAC treated-live WT (n=2) vs Placebo treated-live WT (n=2), NAC-treated dead heterozygous (n=1) vs placebo treated-dead heterozygous (n=1), and NAC treated-null (n=1) vs placebo treated-dead null (n=2). The size of 10 random alveolar spaces were measured in each lung tissue as described in supplementary methods. The unpaired t-test was used to compare the mean size of alveolar spaces of total measured values between NAC treated and placebo. Heterozygous, *Man1a2*^+/-^ ; WT, wild type.

**Supplementary Table 4.** 847 unique differentially expressed genes identified with RNAseq in the lung and liver from NAC-treated pups. These genes are associated with primary ciliary dyskinesia (PCD), CPLANE network, oxidative stress response, glutathione metabolism, Hypoxia response, Lung development, Epithelial tube morphogenesis, Endothelial tube morphogenesis, Man1a2 interactors, N- glycan-biosynthesis and unfolded protein response (UPR).

**Supplemental Table 5.** 297 nodes and numbers of interactions for each node. These nodes represent proteins corresponding to 297 of 847 unique differentially expressed genes in Supplementary Table 4.

**Supplementary Table 6.** Proteins corresponding to 75 differentially expressed genes in a key network cluster shown in Supplementary Figure 7.

**Supplementary Table 7.** Transcription factors among the 297 network nodes and their interactions. Differential expression of these factors is shown in Supplementary Figure 4.

**Supplementary Table 8**. Comparative gene ontology enrichment analysis. Results for 3223 genes involved in fetal human lung development are shown in the Table on the left. Results for *Man1a2* mutant pups are shown in the Table on the right.
